# Marine phytoplankton impose strong selective pressures on *in vitro* microbiome assembly, but drift is the dominant process

**DOI:** 10.1093/ismeco/ycaf001

**Published:** 2025-01-06

**Authors:** Sergio E Morales, Sven P Tobias-Hünefeldt, Evelyn Armstrong, William S Pearman, Kirill Bogdanov

**Affiliations:** Department of Microbiology and Immunology, University of Otago, PO Box 56, Dunedin 9054, New Zealand; MPG Ranch, Florence, MT 59833, United States; Department of Microbiology and Immunology, University of Otago, PO Box 56, Dunedin 9054, New Zealand; Department of Plankton and Microbial Ecology, Leibniz Institute for Freshwater Ecology and Inland Fisheries (IGB), Zur Alten Fischerhuette 2, D-16775 Stechlin, Germany; Department of Microbiology and Biotechnology, University of Hamburg, Ohnhorststraße 18, Hamburg 22609, Germany; NIWA/University of Otago Research Centre for Oceanography, Department of Marine Science, University of Otago, PO Box 56, Dunedin 9054, New Zealand; NIWA/University of Otago Research Centre for Oceanography, Department of Marine Science, University of Otago, PO Box 56, Dunedin 9054, New Zealand; Department of Microbiology and Immunology, University of Otago, PO Box 56, Dunedin 9054, New Zealand

**Keywords:** environmental filtering, phycosphere, iCAMP, ecological selection, phytoplankton blooms, Munida Microbial Observatory Time-Series

## Abstract

Phytoplankton are known ecosystem engineers that modulate ocean community assembly processes, but the universality and extent of their microbiome control remains unclear. We used *in vitro* incubations and 16S ribosomal RNA gene amplicon sequencing to test the influence of Southern and South Pacific oceans dominant phytoplankton on assembly processes and community successions in response to phytoplankton blooms. Phytoplankton grown with reduced-diversity cultures or supplemented with exogenously added microbiomes showed reduced diversity, suggesting environmental filtering. Community profiles were distinct under all culture conditions, further confirming strong selection for specific microbiomes based on phytoplankton. Analysis of core, abundant, and rare organisms in each culture condition showed a conserved response in which core organisms were enriched under conditions of exogenously added phytoplankton. Progression through phytoplankton growth phases selected first for rare and abundant organisms, with increased selection for core members during the exponential phase and relaxing of selection during the death phase, as seen throughout incubations for microbiome-only controls. Surprisingly, selection process quantification identified drift as the dominant process across all conditions and growth phases, with homogenous selection and dispersal limitation accounting for the remainder. Altogether, using Southern Ocean–derived model organisms we confirmed the role phytoplankton play in community assembly but also demonstrated that stochastic processes still predominately drive community selection.

## Introduction

Phytoplankton form the base of the marine food web. Spanning coastal [1] to global [2] waters, phytoplankton account for >40% of marine primary production. Through photosynthesis phytoplankton convert inorganic carbon to organic carbon for use by other organisms, including prokaryotes in their vicinity. The term “phycosphere,” originally described in 1972 [3], is used to define the region surrounding phytoplankton. This area, akin to the plant rhizosphere, is rich in secreted compounds leading to increased bacterial growth [4]. Phytoplankton can also influence microbial communities after death through provision of nutrients [5]. Changes in composition of primary producers are linked to ecosystem scale variations in both carbon export [6] and overall microbial community composition, [7, 8] further indicating a role in prokaryotic community assembly. However, the scale of influence and the role of phytoplankton as community engineers are still under exploration [9].

While phytoplankton are known to associate, or select, for specific microbial communities, this association is expected to be a two-way interaction, with prokaryotes providing nutrients or limiting factors such as vitamins [10]. So, while phytoplankton can provide sources of energy and carbon to oceanic communities, when nutrients are low the associated microbiomes of phytoplankton provide growth benefits from mineralization of nitrogen and phosphorus by their associated microbiomes [4]. This finding implies strong selection pairing specific phytoplankton, and their associated metabolites, to distinct prokaryotic communities under deterministic selection processes like environmental filtering, where the environmental selection of species with similar trait values within communities leads to a reduction in species diversity because not all species can persist. So, under strong selective pressures with low levels of functional redundancy, filtering leads to only a portion of potential species in the landscape or regional pool being suited for the modified conditions. This finding is supported by both culture [2] and field studies [7] linking changes in microbial communities to changes in dominant phytoplankton. These changes and phytoplankton-driven successions are likely controlled in part by environmental filtering through chemotaxis [5, 11, 12]. Direct testing of this concept by using synthetic phycospheres showed predictable bacterial community assembly based on individual metabolites, attributing a strong role for host phytoplankton in controlling bacterial associates [9, 13]. This strong level of control between host metabolism and associated prokaryotes occurs because of the ability of exudates to serve as a single source of nutrition, with coastal and open-ocean bacterial communities receiving organic carbon, nitrogen, and phosphorus directly from a single source [14].

Despite significant progress, reconciling observations made in simplified systems to whole-ocean processes remains a work in progress. Field-scale observations tend to ignore microbial-scale processes and average patterns across large scales that represent the non–phycosphere-influenced ecosystem [15, 16]. In contrast, laboratory-based studies must contend with bottle effects, bias in phytoplankton selection, and relevance of studied organisms to specific locations. In the Southern Hemisphere, long-term data are limited to Antarctica, with only one site with available data regarding long-term microbial composition [17]. This paucity of available data make the Southern Ocean and South Pacific Ocean understudied regions for which limited information is available on the role of phytoplankton in controlling prokaryotic community assembly and related ecosystem processes. Prior research has identified nanoflagellates, dinoflagellates, diatoms, coccolithophores, and cyanobacteria as transiently dominant phytoplankton [18–22]. The dominance of these organisms is spatially and temporally dynamic, with marked changes along the South Subtropical Convergence Province (SSTC) and the Subtropical Front (STF) [23]. Around New Zealand, diatoms can account for 70% to >95% of total cell carbon, but dominance is inconsistent, with nanoflagellates and dinoflagellates reaching high abundances as well. Small-celled diatoms like *Pseudonitzschia*/*Nitzschia* (currently referred to as *Cylindrotheca*) can become the most common diatom taxon in nutrient-replete waters [19, 20]; however, picoplankton (<2 μm), including *Synechococcus*, are the dominant plankton in oligotrophic systems with low productivity [24]. These picoplankton are complemented by other organisms, including more than 45 coccolithophore species, with *Emiliania huxleyi* being the dominant species [18, 21, 22]*.* In these waters, phytoplankton blooms can shift from a diatom-dominated to a pico- and nanoplankton-dominated system mediated by viral blooms and predation that release nutrients back into the ecosystem [25]. These observations suggest that changes in phytoplankton over space and time are in part driving observed changes in community composition [18, 26–30]. Currently, we lack quantification of impacts of phytoplankton on community assembly for relevant organisms in southern waters.

In this study, we tested the impacts of different laboratory-grown primary producers on prokaryotic community assembly and whether their microbial successions display signs of core, rare, and abundant microbial taxa across growth phases and conditions. To do so we utilized two common and abundant Southern Ocean and South Pacific Ocean phytoplankton varieties representing different functional groups (the small-celled pennate diatom *Cylindrotheca closterium* [formerly *Nitzschia* spp.] and the globally ubiquitous coccolithophore *E. huxleyi*). Cultures of each were grown with or without addition of extraneous microbiomes sourced from local waters, and prokaryotic community changes were monitored through 16S amplicon sequencing. We hypothesized that different phytoplankton impose distinct selective pressures, leading to changes in environmental filtering and unique prokaryotic successions in response to primary producer blooms. In this report we provide culture-based evidence for strong filtering leading to distinct communities, primarily by growth phase followed by dominant phytoplankton. However, stochastic processes like drift dominate community assembly.

## Materials and methods

### Experimental set-up

Cultures of *E. huxleyi* and *C. closterium* were originally isolated in December 2016 from mesocosms containing water from Wellington Harbour. Cultures were maintained in 10% Guillard’s (F/2) medium (Sigma). Seawater for the medium was collected from the Portobello Marine Laboratory, UV treated, and prefiltered through a 1-μm filter before being sterilized by microwaving for a total time of 12 minutes (in 3- to 4-minute rounds), with mixing between each round. Nutrient concentrations were amended by addition of 96 μM nitrate and 6 μM phosphate and in the case of *C. closterium* 10 μM silicate.

Two phytoplankton species and microbiome presence were used as experimental variables, yielding five experimental groups (only *E. huxileyi*, *E. huxileyi* + microbiome, only *C. Closterium*, *C. Closterium* + microbiome, and only microbiome [bacterial control]). Investigation of each experimental group was performed in triplicate across four growth phases, yielding a total of 12 bottles per treatment. Each 500-ml culture consisted of f/20 medium supplemented with 50 ml of 0.22- or 2-μm filtered Otago Harbour water as a microbiome inoculum. Phytoplankton inoculum consisted of 1 ml for *C. closterium* or 0.5 ml for *E. huxleyi*. It should be noted that pure phytoplankton cultures were never exposed to antibiotics to create truly axenic cultures, so carryover microbiomes were present.

After all bottles were inoculated, three bottles of each phytoplankton/microbiome mix were designated for sampling at the following time points: lag (starting time point [T_0_]), exponential, stationary, and death. Bottles were incubated at 14°C and exposed to light:dark illumination cycles (12 hours each) at 95 ± 5 μmol m^−2^ s^−1^. All bottles were maintained without aeration but with lids untightened and once a day were gently inverted every 3 seconds for 30 seconds.

Phytoplankton growth was monitored a minimum of every 2 days for average growth measurements. Subsamples were taken after gentle inversions for 30 seconds and dark adapted for 30 minutes, and the *in vivo* chlorophyll *a* (Chl *a*) fluorescence was measured on a Turner 10-AU fluorometer. As measuring chlorophyll in every bottle each day would result in insufficient sample volume by the end of the experiment for all analyses required, only the chlorophyll in the next bottles to be harvested was measured (i.e., following T_0_, *in vivo* Chl *a* was measured in exponential bottles and following the harvest of exponential bottles *in vivo*, Chl *a* was measured in stationary bottles and so on).

### Physicochemical analysis

Bacterial abundance was assessed via flow cytometry following established protocols [31, 32] using a fluorescence-activated cell sorter (Canto II, Benton and Dickinson) with a blue laser (488 nm). In brief, 2-ml samples were glutaraldehyde fixed for a final concentration of 1%, incubated in the dark for 10 minutes, flash-frozen in liquid nitrogen, and kept at −80°C until analysis. Analysis consisted of staining 0.4 ml of sample to a final 1: 10000 Sybr Green I concentration, which was incubated in the dark for 15 minutes. Samples were run at low speed (12 μl per minute) through the cytometer for 2 minutes each, with a maximum of 100 000 events and 800 events per second, and green fluorescence (FL1)–positive events were counted on an FL1 versus side scatter plot using FlowJo10 software. Red fluorescence was measured against FL1 to distinguish Picoalgae from bacteria.

At the time of harvest, following gentle inversion for 30 seconds, 30 ml of culture was filtered through a GF/F filter. The cells on the filter were analysed for *in vitro* Chl *a* and the filtrate was analysed for nutrients (NH_4_^+^/NO_x_/SiO_2_/PO_4_) with a Lachat Quickchem Flow Injection Analysis System at the Portobello Marine Laboratory. *In vitro* Chl *a* was determined by extraction with 90% acetone at 4°C for 20 hours in the dark. Chlorophyll fluorescence was measured before and after acidification with a Turner 10 AU fluorometer that was calibrated with pure Chl *a* from spinach (Merck) [33]. The remaining culture volume (430–480 ml) was filtered through a 0.2-μm polycarbonate filter to collect bacteria for DNA extraction.

### 16S ribosomal RNA gene sequencing and processing

Total community DNA was extracted using a Qiagen PowerSoil® kit following the manufacturer’s protocol but using a Geno/Grinder for 2 × 15 seconds instead of vortexing at maximum speed for 10 minutes. DNA quantity and quality were assessed using a Nanodrop Spectrophotometer (Thermo Fisher Scientific, Waltham, MA, United States). All DNA was stored at −20°C until further use.

Community profiles were generated per the Earth Microbiome Project (Caporaso *et al.*, 2012) [34]. The V4 region of the 16S ribosomal RNA (rRNA) gene was amplified using the 515F (5′-NNNNNNNNGTGTGCCAGCMGCCGCGGTAA-3′) and 806R (5′-GGACTACHVGGGTWTCTAAT-3′) primers. Barcoded samples were loaded onto an Illumina MiSeq (16S) 2 × 150–bp run (Illumina, CA, United States). All sequence data from this study have been deposited in NCBI under BioProject PRJNA1126086.

16S amplicon sequencing reads were quality filtered and assigned to amplicon sequence variants (ASVs) using the dada2 R package (version 1.26) and associated pipeline (Callahan *et al.*, 2016) [35]. Taxonomy was identified with the use of the SILVA rRNA reference database (version 138) using the Ribosomal Database Project naïve Bayesian classifier method (Wang *et al.*, 2007) [36]. Amplicon sequence counts were rarefied a total of 10 times to 30 000 reads per sample using the *rarefy_even_depth* function in the *phyloseq* package, and the average results were used for downstream analysis.

### Statistical analyses and visualization

To test for significant correlations between nutrients and over time we used a Spearman test with the cor.test() function from the stats package (version 4.4.0). Logarithmic response ratio analysis of physicochemical parameters by culture conditions was performed using SingleCaseES [37].

Species richness and Shannon diversity were calculated with the phyloseq [38] estimate_richness function, while evenness was determined using the microbiome package evenness function [36]. Community dissimilarities were determined using the Bray–Curtis distance, with significant changes in community structure or variance tested via analysis of similarity (ANOSIM) and analysis of dissimilarity (ADONIS), respectively. The comparisons were performed at each growth phase within all algal and bacterial treatments and again including all data. Differences in communities were visualized using nonmetric multidimensional scaling (NMDS) plots. ASVs significantly affected by treatments (*P* < .05) were identified using a kruskal_test from the rstatix package [40] for each growth phase separately by comparing the bacterial control (i.e. microbiome only) to other experimental groups.

For ternary analyses, ASVs were grouped into one of three groups (core, abundant, or rare). Core microbes were defined based on the contribution of each ASV to the overall beta diversity of that experimental condition using methods and code developed by Shade and Stopnisek [41] using an elbow cut-off (based on first-order differentiation, see Shade and Stopnisek [41] for an in-depth explanation). Abundant microbes were those microbes which were not core but had a mean abundance of 0.01% or greater, while rare microbes were those with a mean abundance of <0.01%.

Inferences of selective mechanisms shaping community assembly were conducted using phylogenetic bin-based null model analysis (iCAMP; version 1.5.12) and NST (version 3.1.10) packages in R [42, 43]. Phylogenetic trees were produced for these analyses using MAFFT (version 7.505) to align ASV sequences and FastTree (version 2.1.11) with a GTR-CAT model and the FFT-NS-2 option enabled and a maximum of 10 iterations. Phylogenetic trees were inspected for outlier taxa based on branch lengths (outliers were classified as those with branch lengths >1.1, Fig. S1), following methods and code developed by Gundersen and Vadstein (2024) [44], which resulted in 6 ASVs being removed. We ascertained the presence of phylogenetic signal within our data following methods by Gundersen and Vadstein (2024), based on correlations between the environmentally weighted abundance of ASVs and phylogenetic distances (Fig. S2). Environmental conditions for this analysis were generated by using factor analysis of mixed data (FAMD), as implemented in FactoMineR [45], using culture type (e.g. bacteria, etc.), experimental phase (lag, stationery etc.), bacterial status (added versus not added), and primary producer as categorical variables. Physicochemical variables were treated as continuous variables for the FAMD. Each set of iCAMP or taxonomic NST (using Jaccard dissimilarity) analyses were conducted separately for each experimental group—with meta-communities defined as the overall community within each experimental group (e.g. phytoplankton only versus phytoplankton + microbiome). iCAMP/NST analyses used a bin size limit of 20 taxa, with phylogenetic differences between communities determined based on beta mean nearest taxon index with a confidence-based cut-off (0.975). Per sample nearest taxon index (NTI) values were calculated with the NTI.p R function and are reported in the supplementary material.

## Results

### Physicochemical profiles change by growth phase and culture type

**Figure 1:**
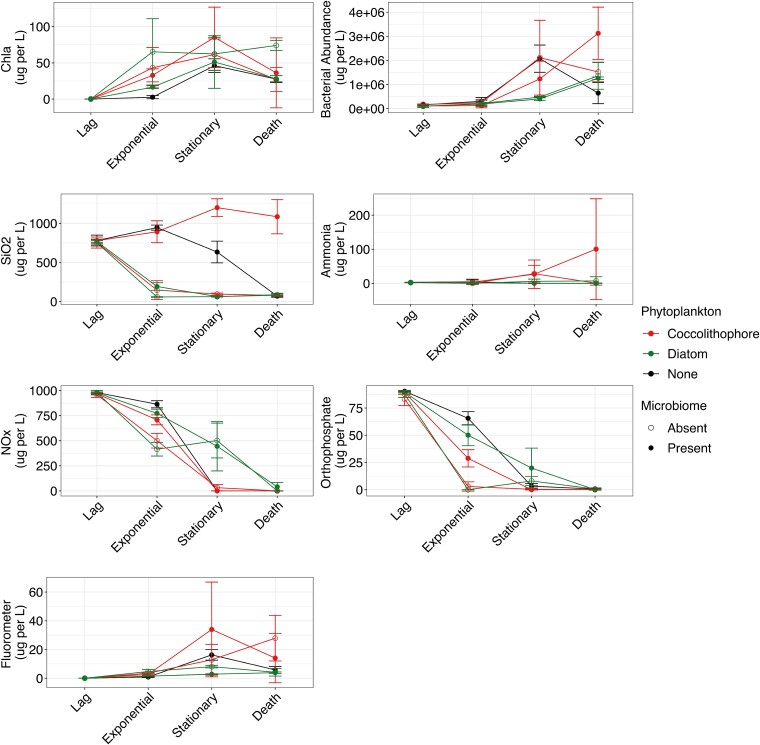
Changes in physicochemical parameters across culture conditions and growth phase. Panels show concentration of chlorophyll α (Chl α), total bacteria, SiO_2_, ammonia, NO_x_, and orthophosphate (OP) based on triplicate cultures. Colors indicate exogenously added phytoplankton, and shape indicate presence (solid circle) or absence (open circle) of exogenously added microbiomes in the form of seawater. Error bars represent standard deviation. Statistics are provided in Table 1 for significant differences between conditions.

We monitored physicochemical conditions in cultures over a 700-hour (~30 day) period (Fig. 1 and Fig. S3, Table 1). A phytoplankton bloom (determined by depletion of N/P and increases in Chl-a) was detected in all cultures, indicating the presence of phytoplankton in source water for microbiome recruitment. Effects across all measured parameters showed distinct responses to culture conditions (effect sizes are summarized in Fig. S4). Across all parameters measured we observed a highly dynamic system over time, with phytoplankton growth phase leading to changes in physicochemical conditions. All culture conditions led to a chlorophyll *a* bloom (mean 2.9 × 10^6^% increase) and increases in bacterial abundance (mean 1238% increase), though timing of changes differed across culture conditions. Concentration of SiO_2_ decreased rapidly in coccolithophore-only cultures from the lag to exponential growth phase (80.6% decrease), which was not the case when exogenously added microbiomes were present (14.6% increase), which was the only condition with continuously increasing SiO_2_ concentrations across the entire timeframe. In controls with no added phytoplankton, SiO_2_ values remained high, with a drop (92.7% decrease) from the exponential to the death phase, suggesting an unknown phytoplankton bloom utilizing the resource.

**Table 1:** Significant monotonic nutrient changes independent of culture conditions using Spearman tests.

**Nutrient/variable**	**Secondary nutrient**	**rho**	***P*-value**
Hours	Intracellular Chl *a*	0.762	.028
Hours	Bacterial Abundance	0.976	<.001
Hours	NO_x_	−0.952	<.001
Hours	Orthophosphate	−0.952	<.001
Hours	Spectrometer values	0.905	.002
Intracellular Chl *a*	Bacterial Abundance	0.738	.037
Intracellular Chl *a*	SiO_2_	−0.810	.015
Intracellular Chl *a*	NO_x_	−0.786	.021
Intracellular Chl *a*	Orthophosphate	−0.833	.010
Intracellular Chl *a*	Ammonia	−0.762	.028
Intracellular Chl *a*	Spectrometer values	0.810	.015
Bacterial abundance	NO_x_	−0.905	.002
Bacterial abundance	Orthophosphate	−0.905	.002
Bacterial abundance	Spectrometer values	0.881	.004
SiO_2_	Ammonia	0.952	<.001
NO_x_	Orthophosphate	0.952	<.001
NO_x_	Spectrometer values	−0.976	<.001
Orthophosphate	Spectrometer values	−0.929	<.001

Sample (*n* = 60) nutrients were compared irrespective of phytoplankton/exogenous bacteria status, to determine significantly correlated nutrients and changes over time.

Ammonia (NH4^+^) levels remained stable for all cultures, but NO_x_ (mean 33% decrease) and orthophosphate (OP; mean 67.1% decrease) concentrations decreased rapidly, driven by phytoplankton increases (mean 3.4 × 10^5^ increase). NOx and OP decreases were fastest from lag to exponential growth phases in cultures lacking additional external microbiome sources (mean 52.7% NOx and 98.2% OP), while microbiome-only cultures had the slowest decline (mean 23.8% NOx and 55.9% OP) consistent with having the most delayed chlorophyll increases. Consumption of OP, independent of exogenous microbiome addition, was fastest in the larger sized coccolithophore cultures (82.3%) compared to the smaller diatoms (72%).

### Beta and alpha diversity are modulated by growth phase and culture type

**Figure 2:**
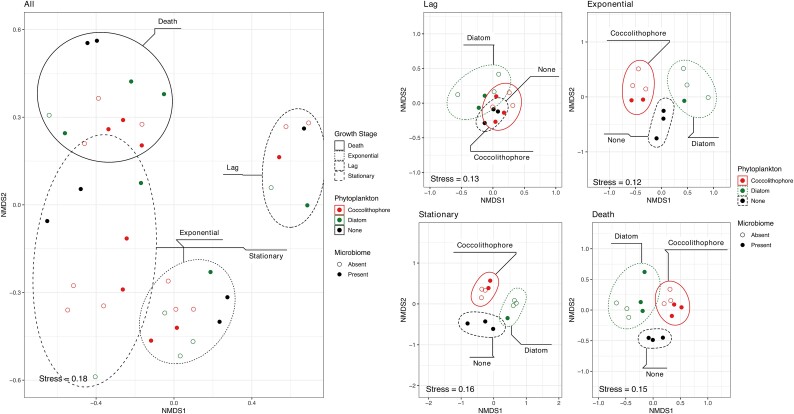
Change in prokaryotic microbial beta-diversity in response to treatment and growth phase. Nonmetric multidimensional scaling (NMDS) analysis plot using Bray–Curtis distance show community response across all samples or when processed separately by growth phase. Ellipses depict the 95% confidence interval. Statistics are provided in Table 2.

**Figure 3:**
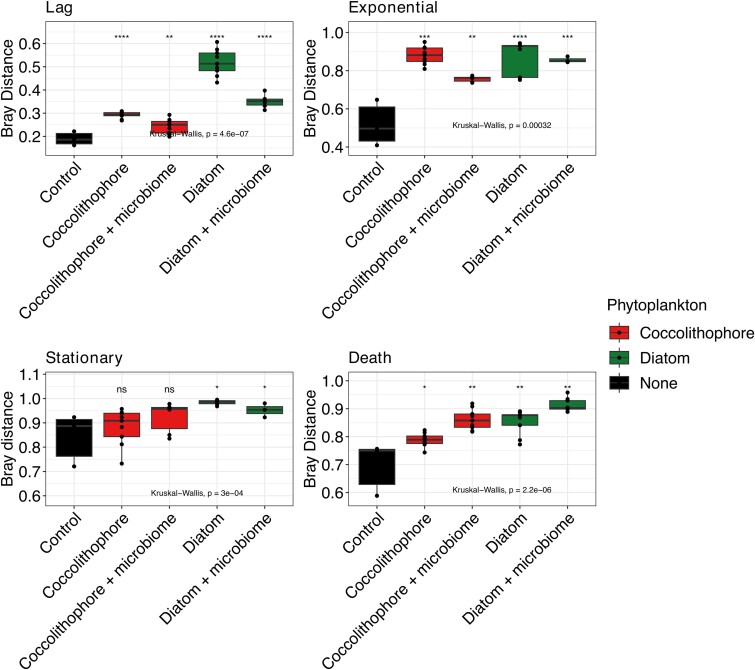
Observed Bray–Curtis dissimilarity of prokaryotic community composition between treatments at each growth stage. For each plot, treatments were compared to control only seawater samples. Global means was calculated using Kruskal Wallis test. Individual comparisons against the control were performed using a *t*-test.

Microbial communities in all cultures were profiled by 16S rRNA gene amplicon sequencing, with 53 samples retained after rarefication. Growth phase was the main driver of community dissimilarity (Table 2), followed by culture type (Fig. 2) despite both being significant (ANOSIM and ADONIS *P* < .01). To control for differences driven by growth phase, samples were analyzed separately according to growth phase to identify the impact of culture condition (Fig. 2–3). Evidence of significant changes (ANOSIM and ADONIS *P* < 0.01) from the no phytoplankton control were observed at all growth phases with the least difference seen during stationary phase. This is likely due to increased variance at that time. Combined with results from NMDS analysis it shows distinct communities for each treatment/growth phase combination indicative of unique ecosystem filtering conditions for each. Diatoms had the biggest impact based on observed dissimilarity. The impact of phytoplankton is also seen across alpha diversity metrics with observed richness values (Fig. S5 and Fig. S6). All cultures showed decreased richness at the last sampling time, again suggesting strong selective pressure by culturing in presence of phytoplankton.

**Table 2:** *R* statistic and significance testing for shifts in community composition at different growth phases based on culture type.

**Growth phase**	**Test**	**Variable tested**	**R**	***P* value**
All	ANOSIM	Culture type	0.359	.001
All	ADONIS	Culture type	0.222	.001
All	ANOSIM	Growth stage	0.573	.001
All	ADONIS	Growth stage	0.222	.001
Lag	ANOSIM	Culture type	0.874	.001
Lag	ADONIS	Culture type	0.781	.001
Exponential	ANOSIM	Culture type	0.793	.001
Exponential	ADONIS	Culture type	0.795	.001
Stationary	ANOSIM	Culture type	0.650	.002
Stationary	ADONIS	Culture type	0.623	.001
Death	ANOSIM	Culture type	0.846	.001
Death	ADONIS	Culture type	0.638	.001

### Change in dominant phytoplankton results in modified environmental filtering

**Figure 4:**
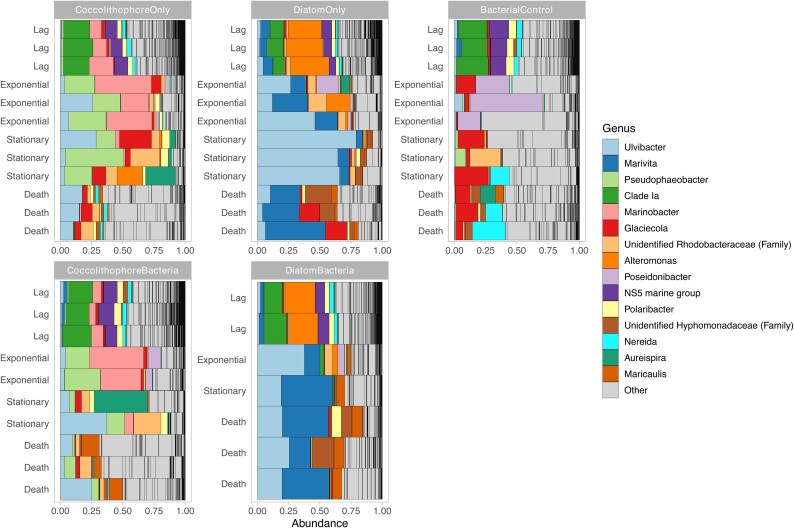
Relative abundance of dominant microbiome taxa across culture conditions colored at the genus level.

To identify taxonomic rearrangements indicative of changes in environmental filtering we compared changes in community composition across culture condition and growth phase. While community profiles were generally represented by similar phyla (Fig. S7), their relative abundance was affected. Changes in composition became more apparent as taxonomy level was lowered to Order level (Fig. S8). When profiles were compared across dominant genera, unique community profiles and successions for each culture condition were observed (Fig. 4). For control cultures with no extraneously added phytoplankton key changes in mean relative abundance included time dependent blooms of *Poseidonibacter* (peaked at 30% relative abundance in exponential stage), *Glaciecola* (10% at stationary and 11% at death stage), and *Nereida* (12% at death stage). For Coccolithophore cultures blooms of *Marinobacter* and *Pseudophaeobacter* were prominent in early growth phases, with higher abundances under conditions without added microbiomes (29% and 20% at exponential phase, respectively). For diatom cultures *Ulvibacter* and *Alteromonas* were the most recognizable genera. Across all conditions, *SAR11 Clade Ia* was universally present but displayed variable population sizes with the highest abundances associated with coccolithophore cultures, followed by control and diatoms cultures. Members of the *Ulvibacter* and an unknown chloroplast were also universally detected but with variable population sizes across culture conditions and growth phase.

**Figure 5:**
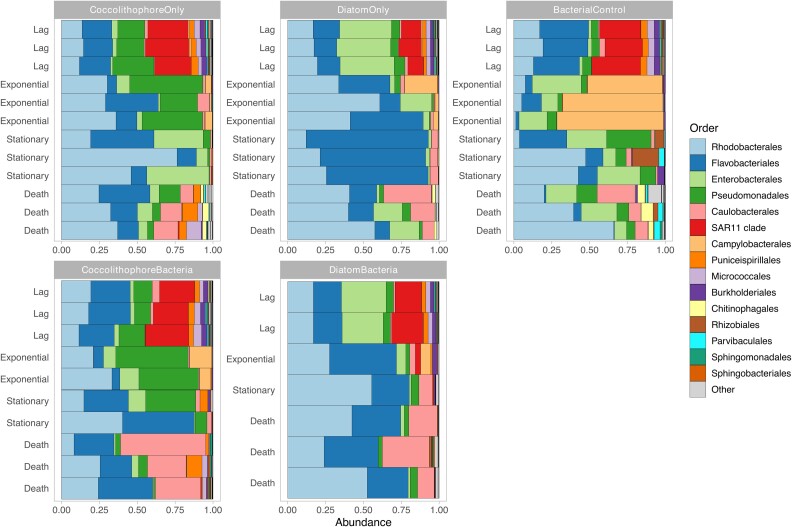
Significantly affected taxa in response to culture conditions grouped at order level. Taxa were identified using a Kruskal-Wallis test for each growth phase separately by comparing control profiles to those with phytoplankton present. Only top 15 most abundant groups shown with remainder shown as other.

To provide a quantitative assessment of taxonomic differences, we used the rstatix package and identified taxa showing significant differences (Kruskal-Wallis *P* < .05) from control cultures for each growth phase (Table S1). Then, all significantly affected taxa were compiled and its total abundance was used to show taxonomic composition. It confirmed unique community profiles and successions for each culture condition across all taxonomic levels (Fig. 5 and Fig. S9–11). Both presence/absence of source microbiomes, as well as dominant phytoplankton, impacted microbiome composition. Representative phylogenetic orders for controls included *Campylobacterales* and *Burkholderiales*, while *Pseudomonadales* dominated within coccolithophore cultures and *Enterobacterales* as well as *Ricketsiales* were predominant under diatoms.

### Culture conditions and time drive shift in selective pressure and community structure

**Figure 6:**
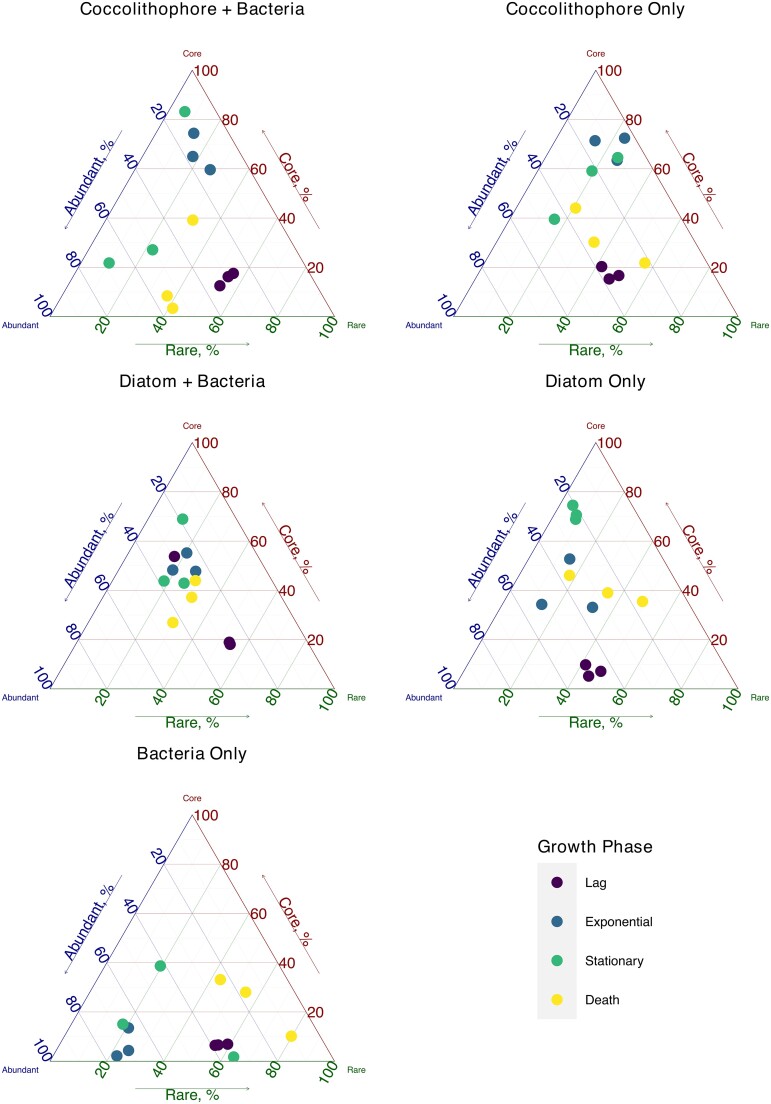
Ternary plot of the distributions of microbial communities (color coded by growth phase) for each culture condition. Core microbes were defined based on contributions of towards overall community beta diversity, and abundant microbes were defined as those with a mean abundance ≥0.1%.

We classified ASVs under each culture condition as either core, abundant, or rare based on average abundance and examined their relative contributions to community structure (Fig. 6). A higher prevalence of core organisms was detected in the presence of exogenously added phytoplankton, but changes in the community were distinct across all cultures. During the start of incubations (lag phase) there was a higher contribution of both rare and abundant ASVs. Transition into the exponential phase led to increases in core ASVs, with the proportion of ASVs highest in the presence of coccolithophores, although core ASV numbers increased during the stationary phase in diatom cultures, potentially indicating a delayed response attributable to decreases in core ASVs in coccolithophore cultures during this same period and return to starting conditions during the death phase. A similar trend was observed in diatom cultures, which suggested a conserved response during community succession. In contrast, changes in microbiome-only controls remained more stable, with no increase in core ASVs.

**Figure 7:**
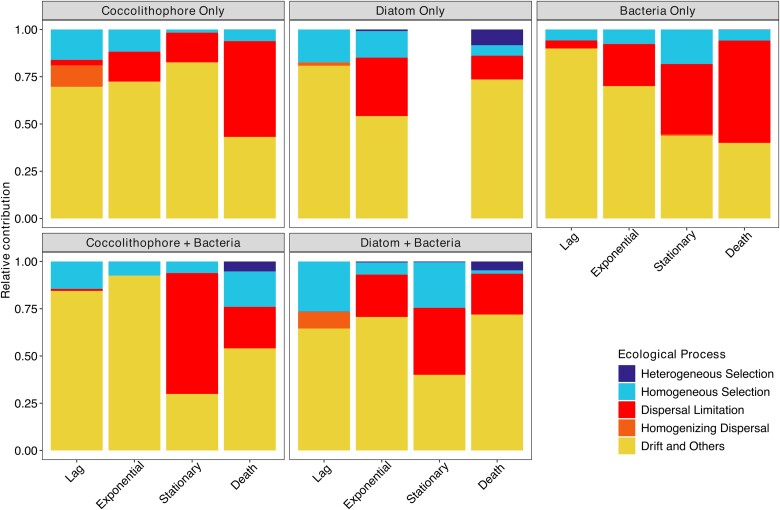
The relative influence of different ecological factors to community assembly under culture conditions throughout the phytoplankton growth phases.

To determine the dominant selective processes impacting community assembly we utilized the iCAMP framework (Fig. 7). Across all conditions stochastic processes accounted for most of the observed changes, although selection processes changed over time and culture conditions. Under microbiome-only controls, selection changed with time shifting from a drift-driven system to dispersal limitation. In contrast phytoplankton cultures did not show as clear a transition across growth phases, with distinct patterns under each time/culture condition. Deterministic processes contributed the most during lag phase across all phytoplankton cultures, but the extent of contribution was unique for each culture. To confirm these observations, we also calculated NST and NTI (Fig. S12–13), which corroborated the observed trends.

## Discussion

It is well accepted that phytoplankton identity can have an impact on recruitment of prokaryotic communities and play an important role in shaping the global marine microbiome [4, 46]. Despite this, direct evidence of the role of phytoplankton in microbiome assembly across all major ocean regions is still lacking. Some studies found that bloom events with different proportions of diatoms and cyanobacteria did not result in distinct bacterial communities [47]. However, advances in sequencing approaches and bioinformatics have progressed to the stage where minor variations in both population size and community structure are now detected, [48] but evidence to confirm the role of phytoplankton as ecosystem engineers for prokaryotic communities is still needed [9, 12, 49]. In this study we tested the ability of Southern Ocean phytoplankton to control prokaryotic community assembly.

In summary, we observed distinct microbiomes dependent on phytoplankton presence and identity, coupled with the identification of stochastic ecological processes as the key factor in assembling these communities. These changes were associated with differences in observed nutrients that suggest a role for ecosystem engineering (i.e. distinct conditions as a result of the growth of different primary producers). Microbiome addition led to increases in recycling of nutrients. Bacterial abundance was positively correlated to chlorophyll concentration (Table 1), with increases in phytoplankton leading to increases in bacterial abundance, suggesting the likelihood that prokaryotes were growing due to DOC released by phytoplankton into the medium. When microbiomes were included, a delay in the collapse of NOx and OP stocks was observed, which suggested that the microbiomes for these two phytoplankton may increase resource provisioning through remineralization, as seen previously [4, 46, 50, 51]. However, unlike prior studies with similar findings, in the present study we did not observe a difference in maximum cell density for hosts [52].

Impacts of phytoplankton extended beyond physicochemical parameters, with significant changes to both alpha- and beta-diversity (Figs 2 and S3–4). Compared to controls, effects on alpha-diversity were small but consistent across all phytoplankton cultures displaying evidence of environmental filtering. Across all cultures, observed richness decreased over time, with control samples retaining the highest richness, findings indicative of selective pressure from the host but significant differences between phytoplankton. These observations suggest a conserved trait across phytoplankton to change community structure, in this case resulting in lowered richness [53, 54].

Changes in beta diversity were stronger than those observed for alpha metrics, resulting in distinct prokaryotic communities for each culture combination. This finding is consistent with predictions and experimental data [55, 56] and may reflect changes in available metabolites across culture conditions [13] potentially linked to nutrient availability [14, 57]. The results are in line with other observations attributed to metabolic variability [58, 59]. For the model phytoplankton *Phaeocystis*, metabolites changed from free amino acids to sugar alcohols, mono- and disaccharides, and finally to free fatty acids, sterols, and terpenes as blooms progressed from exponential to late stationary phase, [60] providing a probable mechanism for observed prokaryotic community changes over time.

In the current study, while phytoplankton impacts were constant across all growth phases, differences were highest during early stages (lag and exponential), with distinct dominant taxa detected under each culture condition. In bacterial control cultures with no phytoplankton added, *Poseidonibacter*, *Glaciecola*, and *Nereida* had strong blooms in the transition from exponential to death phase. However, blooming genera within phytoplankton cultures in our study showed distinct patterns with coccolithophore cultures rich in the previously identified associated genera *Marinobacter* [61] and *Pseudophaeobacter* [62], the latter associated with stationary-phase cultures. This selection for *Roseobacter*-like organisms, such as *Phaeobacter* species, can mediate the turnover of aging phytoplankton cells [63, 64]. *Within diatom cultures*, *genera associated with diatoms*, *including Ulvibacter* [65, 66] *and Alteromonas*, [67] stood out as abundant. Not surprisingly SAR11 Clade Ia (the most abundant and most studied SAR11 ecotype [68–70]), was found in all conditions but showed preference for coccolithophore cultures. The SAR11 clades Ia and Ib seem to be less responsive to filtering and bloom stages [71], although in the present study they were primarily associated with lag-phase samples but were also found in controls and in lower proportions within diatom cultures.

The observed enrichment of certain prokaryotic taxa lends support to phytoplankton serving as engineers of marine microbiomes through environmental filtering and provide evidence of a probable mechanism at play globally but with strains specific to an understudied part of the world. *Emiliana huxleyi* [72] are among the most important phytoplankton due to their overwhelming abundance during blooms, which drastically alter carbon-cycling dynamics at local and global scales. *Emiliana huxleyi* blooms are associated with prokaryotic communities dominated by SAR11, Marine group II *Euryarchaeota*, and *Rhodobacterales*, with postbloom conditions enriched for *Flavobacteriaceae* and *Pseudoalteromonadaceae*, with communities in *E. huxleyi* blooms highly similar to one another [73]. While the relevance of these organisms for the host is not fully understood, some taxa (e.g. *Sulfitobacter*) may serve to protect against pathogenic bacteria [74].

The diatom *Cylindrotheca* genus (formerly *Nitzschia*/*Pseudo-nitzschia*) is one of the largest diatom genera and is globally distributed [75]. Diatoms in general can control large proportions of primary productivity and also associate with specific microbiomes depending on diatom species, [2] with predictions ranging from mutualistic to commensal or parasitic relationships [76]. As with other hosts, diatom metabolite profiles can select for specific microbiome associations through provision of structurally diverse nitrogen compounds [77]. Marine diatoms commonly co-occur with members of the *Proteobacteria* and *Bacteroidetes* in laboratory cultures and some natural blooms, with the *Alphaproteobacteria* (*Sulfitobacter*, *Roseobacter*, *Ruegeria*, and *Erythrobacter* genera), *Bacteroidetes* and *Betaproteobacteria* being amongst the most abundant [2].

In general, a building consensus supports phytoplankton as direct regulators of microbial community assembly. Although they select for distinct microbiomes, different phytoplankton share a certain level of taxonomic consistency [78], in essence selecting for unique strains within closely related taxa (e.g. different species from *Alphaproteobacteria*). This taxonomic consistency was observed in our study when microbiomes were explored at higher taxonomic levels, leading to consistent detection of certain groups. Our findings also align with observations in natural blooms in which initial blooms are dominated by diatoms followed by smaller phytoplankton, resulting in prokaryotic variation co-occurring with shifts observed at time scales ranging from days to weeks. These observations suggest that the prokaryotic communities are more responsive to phytoplankton dynamics than to environmental factors [7]. While direct evidence is not always available for field studies, a probable mechanism determined through pure culture studies suggest dissolved organic matter and other metabolites also shape Southern Ocean communities [54, 66]. This finding implies that dominant phytoplankton blooms could prime ecosystems for different successions accounting for year-to-year variance in microbiomes. Evidence indicates that prokaryotes receive a large (>50%) proportion of their needed carbon directly from phytoplankton [4] but each microbe may target different substrates, allowing for niche partitioning. This form of environmental filtering through phytoplankton is a mechanism that may have an impact on ecosystem scale processes like carbon cycling by controlling shifts in dissolved organic matter as microbes partition resources, [79] ultimately impacting C-cycling dynamics. This partitioning of resources by microbes leads to selection via different scenarios from resource competition to resource partitioning for specific metabolites [80].

Some critical limitations are highlighted in our study, consistent with prior research. First, our study relied on cultured phytoplankton that were not axenic, thus they were added to our microcosms with a carryover microbiome. When introduced to a new microbiome, the established host microbiome may have an advantage due to a longer acclimatization period with the host. However, in a natural setting algae holobionts are not sterilized prior to changes in community shifts, so selection always occurs within the context of secondary successions. Our experimental system mimics that process, which while reducing the ability to directly attribute cause/effect due to selection better reflects the conditions encountered under natural scenarios. In our study, controls are represented by absence of introduced cultured phytoplankton in order to see changes in microbiomes in the absence of a strong selective pressure imposed by phytoplankton blooms. Further, the use of microcosms leads to well-documented bottle effects [81–83]. In our study the conserved response (decreased observed species; Fig. S6) suggests a strong bottle effect across all treatments, including those with exogenous phytoplankton, indicating that cultured phytoplankton are not experiencing any benefit from prior acclimatization through culturing and are equally impacted. This effect is further supported by the beta dispersion across treatments by time (Fig. 3), which showed control treatments with no phytoplankton having the lowest variance in dissimilarity. Together these findings suggest that despite the known biases associated with our set-up, we still observe strong selection as a result of changes in dominant phytoplankton.

Although our study lends support to the role of phytoplankton in mediating deterministic selection of marine microbiomes, quantification of both deterministic and stochastic processes indicated that stochastic processes, dominated by drift, were the primary driver of community assembly. Deterministic processes have been historically easier to test and are a leading factor in a hypothesis to explain patterns observed from community profiling [84–87]. However, recent work in free-living [88–90] and host-associated [91] microbiomes suggests that stochastic processes may dominate in marine systems, with drift accounting for a large proportion of the selection. This theory aligns with the idea of a selection continuum [92, 93] where both processes contribute to community assembly with changing conditions over time and space leading to tradeoffs between them. In our study system several conditions may have led to a high contribution of stochastic processes. One possible reason is that while the microbes in the different cultures exhibit distinct communities, the within-treatment variability is high, reducing the contribution attributed to selection from the host. A second, and likely higher contributor, is the limited diversity and high role of the rare biosphere. The use of small microcosms inoculated with seawater means they receive a reduced diversity inoculum compared to what is normally used for community profiling (on average > 500 mL of filtered seawater for standard community profiling). This limitation is exacerbated by the large contribution of the rare biosphere, with high variability in detected rare organisms that may result in different processes dominating community assembly for rare versus abundant or common microbes.

Through the use of Southern Hemisphere –specific strains of globally important model organisms, we provide strong evidence of environmental filtering through phytoplankton blooms and a strong role for stochastic processes (i.e. drift). Physicochemical analyses suggest that microbes may assist in nutrient remineralization, benefitting their host through nutrient provisioning and thus prolonging bloom conditions. Further, we show that each host selects for distinct communities, and a shift in selection processes is seen based on culture conditions and growth phase. These findings are supported by prior work and predictions but also provide direct evidence for the Southern Ocean and also continue to build the importance of primary producers, a concept exploited through Longhurst provinces [94–96] based on limits to primary production and parameters that influence phytoplankton growth. Remaining gaps include the level of control that is directly or indirectly attributed to phytoplankton, and how the selection differs across hosts, both closely related (strain to species level variability) and taxonomically diverse.

## Supplementary Material

Supplemental_Figures_ycaf001

Tables_ycaf001

## Data Availability

The 16S amplicon sequencing data are available in the *NCBI SRA Database* (BioProject PRJNA1126086). The OTU table, R scripts, and input files are available on GitHub (https://github.com/k-bogdanov/marine-phytoplankton-2024).
