# Supplementary material for: Marine phytoplankton impose strong selective pressures on in vitro microbiome assembly, but drift is the dominant process
Source: ISME Commun. 2025 Jan 6;5(1):ycaf001. doi: 10.1093/ismeco/ycaf001 (PMC11843096; doi:10.1093/ismeco/ycaf001)
Supplement: Supplemental_Figures_ycaf001 [file supplemental_figures_ycaf001.pdf]

Supplemental Figures

Marine phytoplankton impose strong selective pressures on in vitro microbiome assembly, but drift is the dominant process

Sergio E. Morales[*1,2], Sven P. Tobias-Hünefeldt[1,3,4], Evelyn Armstrong[5], William S. Pearman[5], Kirill Bogdanov[1]

[1]Department of Microbiology and Immunology, University of Otago, PO Box 56, Dunedin 9054, New Zealand

[2] MPG Ranch, Florence, MT 59833, USA

[3] Department of Plankton and Microbial Ecology, Leibniz Institute for Freshwater Ecology and Inland Fisheries (IGB), Zur Alten Fischerhuette 2, D-16775 Stechlin, Germany

[4] Department of Microbiology and Biotechnology, University of Hamburg, Ohnhorststraße 18, Hamburg 22609, Germany

[5]NIWA/University of Otago Research Centre for Oceanography, Department of Marine Science, University of Otago, PO Box 56, Dunedin 9054, New Zealand

***Correspondence:**

Dr. Sergio E. Morales, University of Otago, Department of Microbiology and Immunology, 720 Cumberland Street, North Dunedin, Dunedin 9054, New Zealand

Email: sergio.morales@otago.ac.nz, Phone: + 64 3 479 3140

Running title: Phytoplankton driven environmental filtering

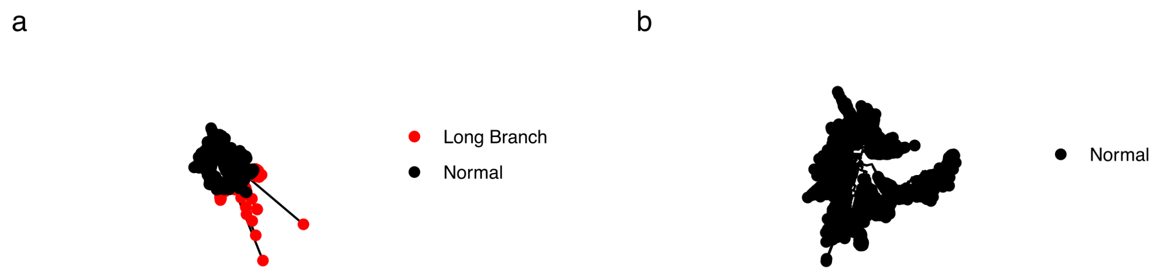

Figure S1 Phylogenetic trees for microbial ASVs before (a) pruning of outlier taxa, and after (b) removal of outlier taxa based on a branch length of > 1.1.

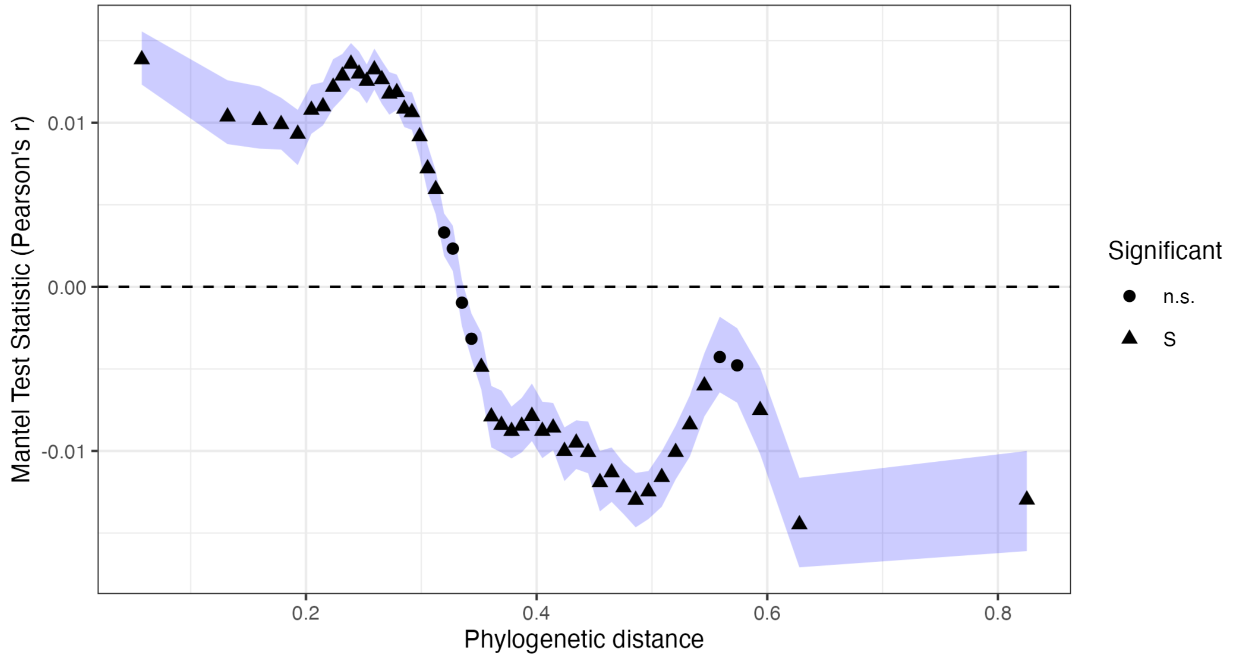

Figure S2 Correlation between environmentally weighted abundance of ASVs and phylogenetic distances.

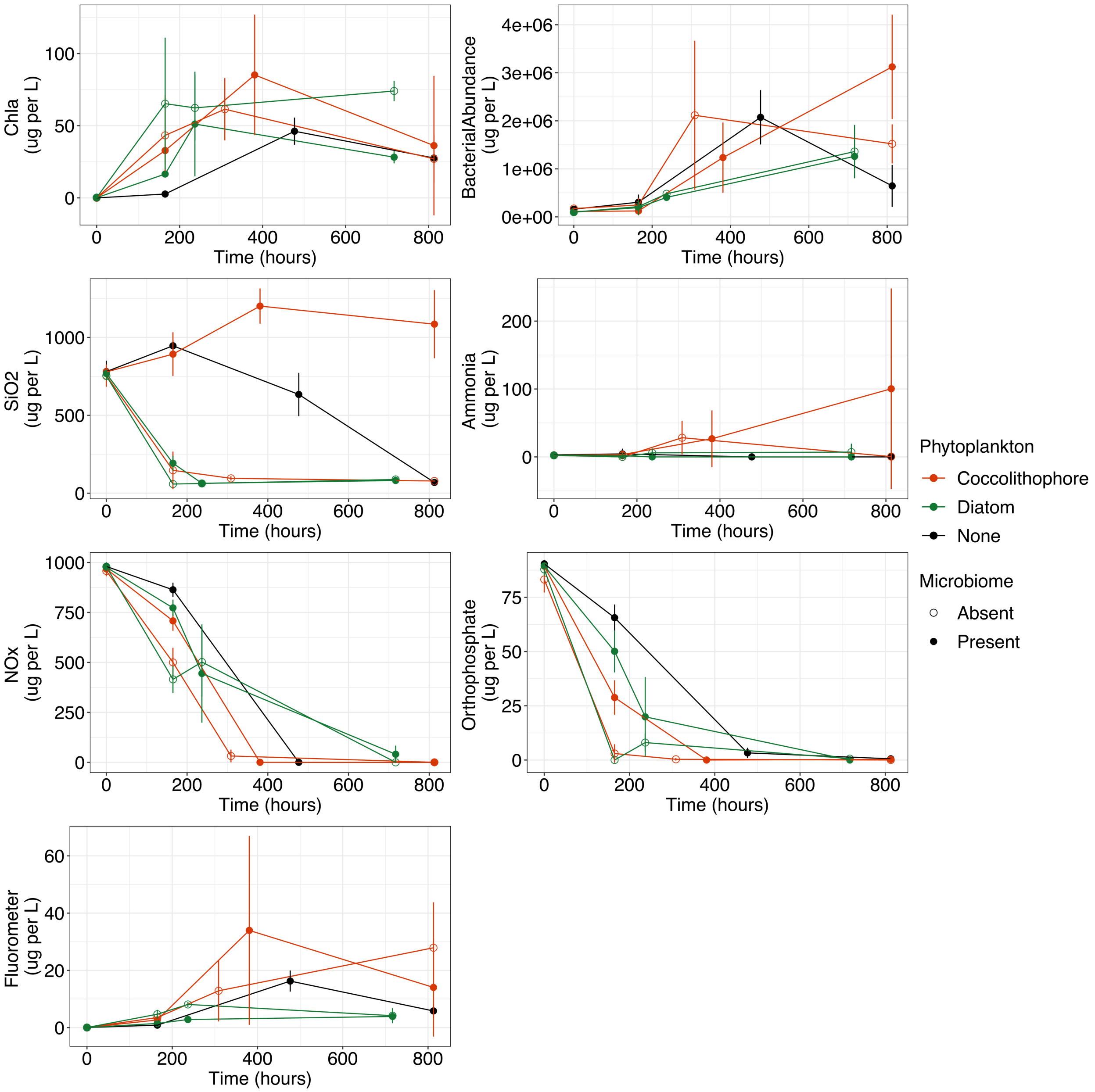

Figure S3 Changes in physicochemical parameters across culture conditions and time.

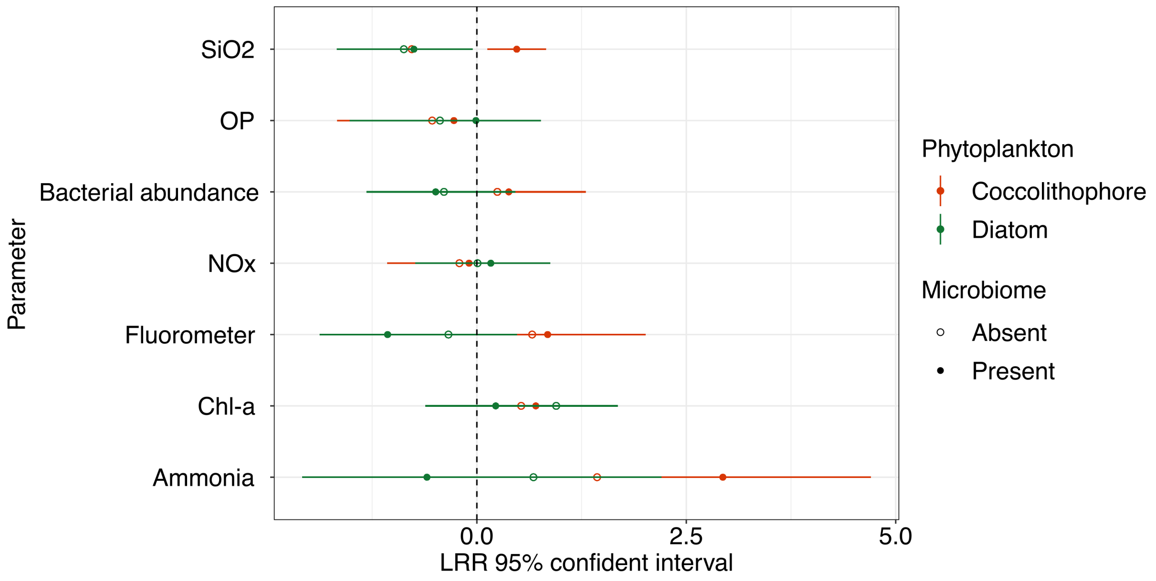

Figure S4 Logarithmic response ratios (LLRs) of statistically affected physicochemical parameters. The response ratio is defined as the value in cultures with added phytoplankton divided by the value in control (no phytoplankton) cultures. Error bars represent 95% confidence intervals. Colors indicate exogenously added phytoplankton, and shape indicate presence (solid circle) or absence (open circle) of exogenously added microbiomes in the form of seawater.

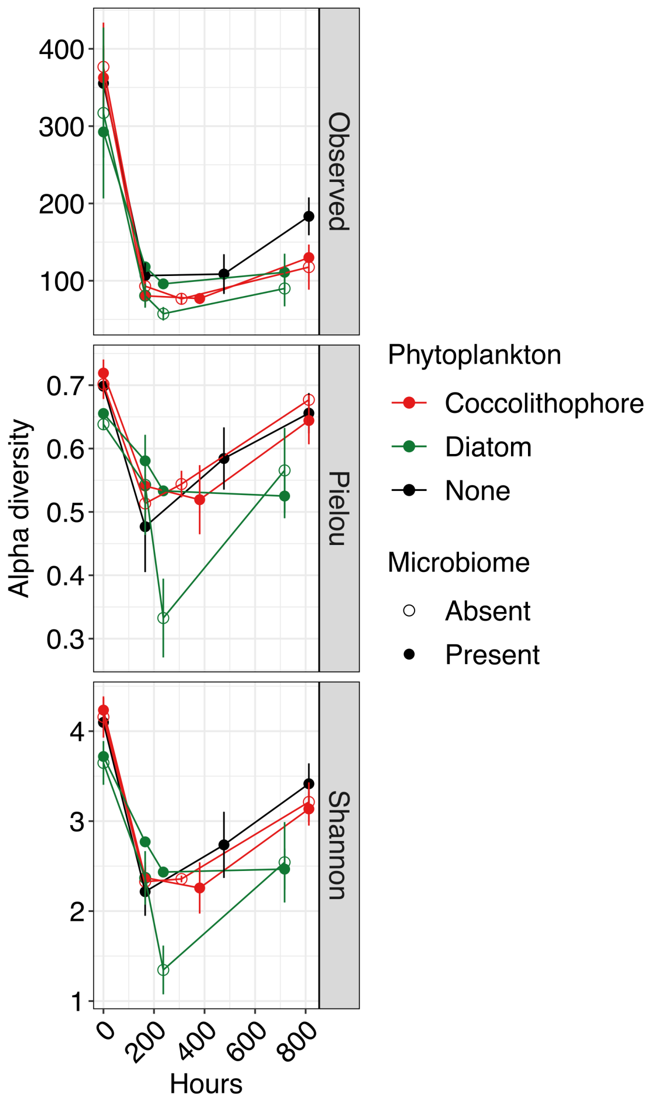

Figure S5 Changes in alpha diversity across culture conditions and time.

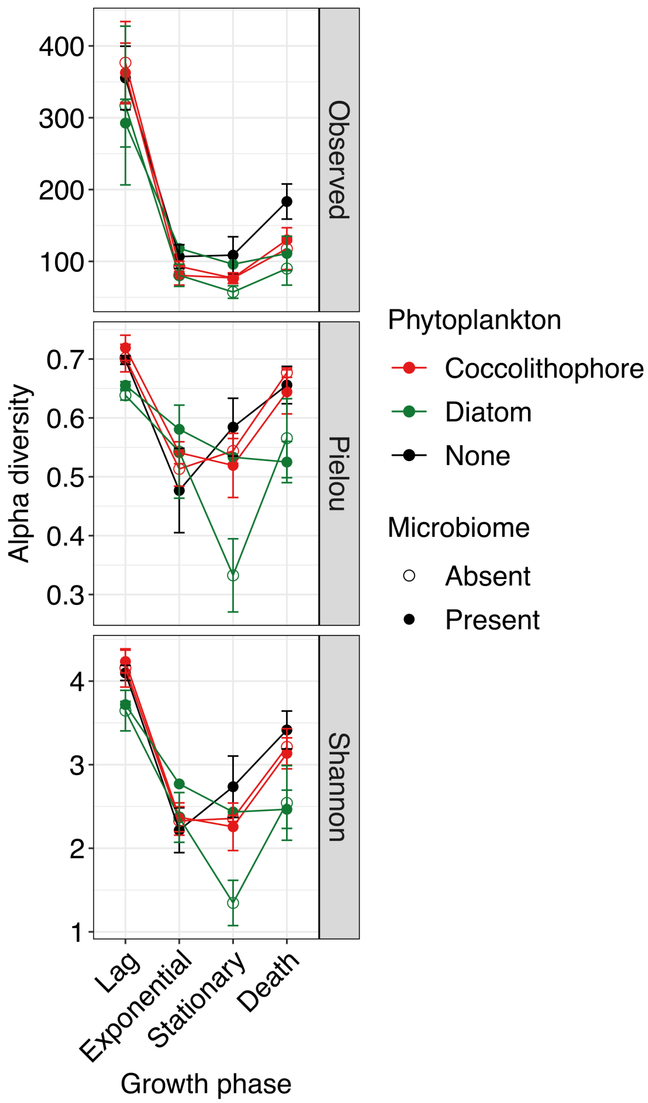

Figure S6 Changes in alpha diversity across culture conditions and growth phase

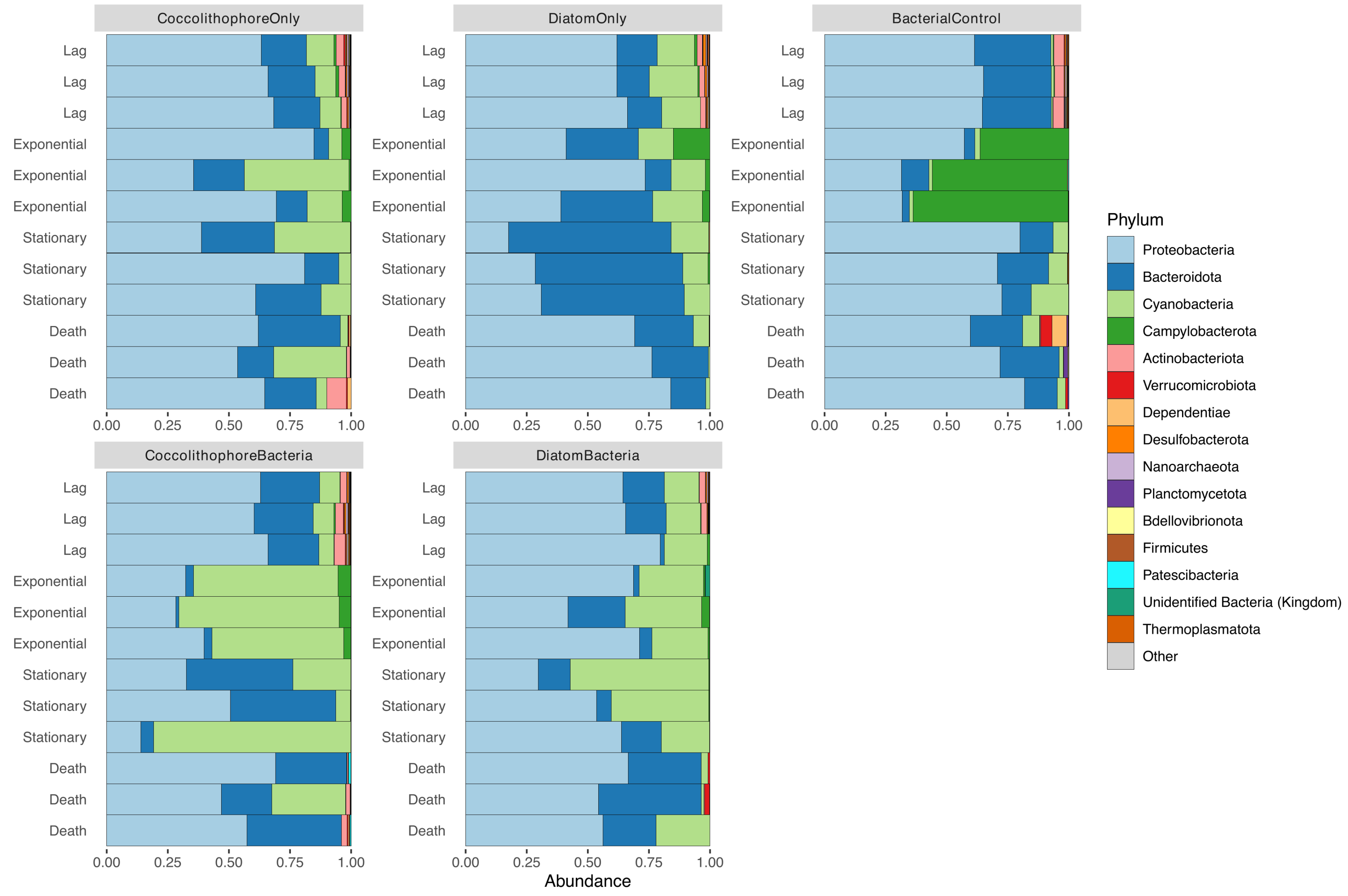

Figure S7 Relative abundance of dominant microbiome taxa across culture conditions colored at Phylum level.

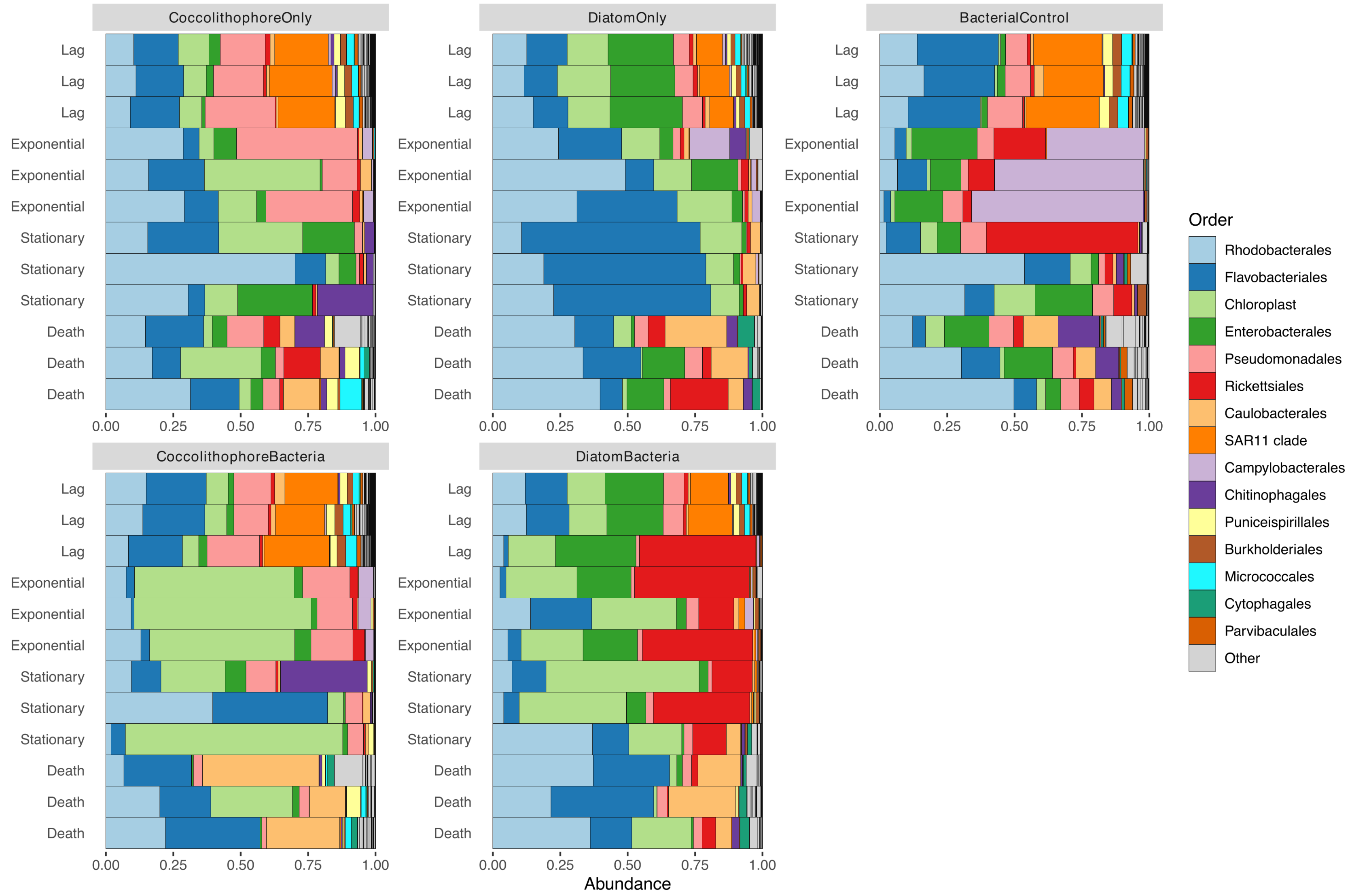

Figure S8 Relative abundance of dominant microbiome taxa across culture conditions colored at Order level.

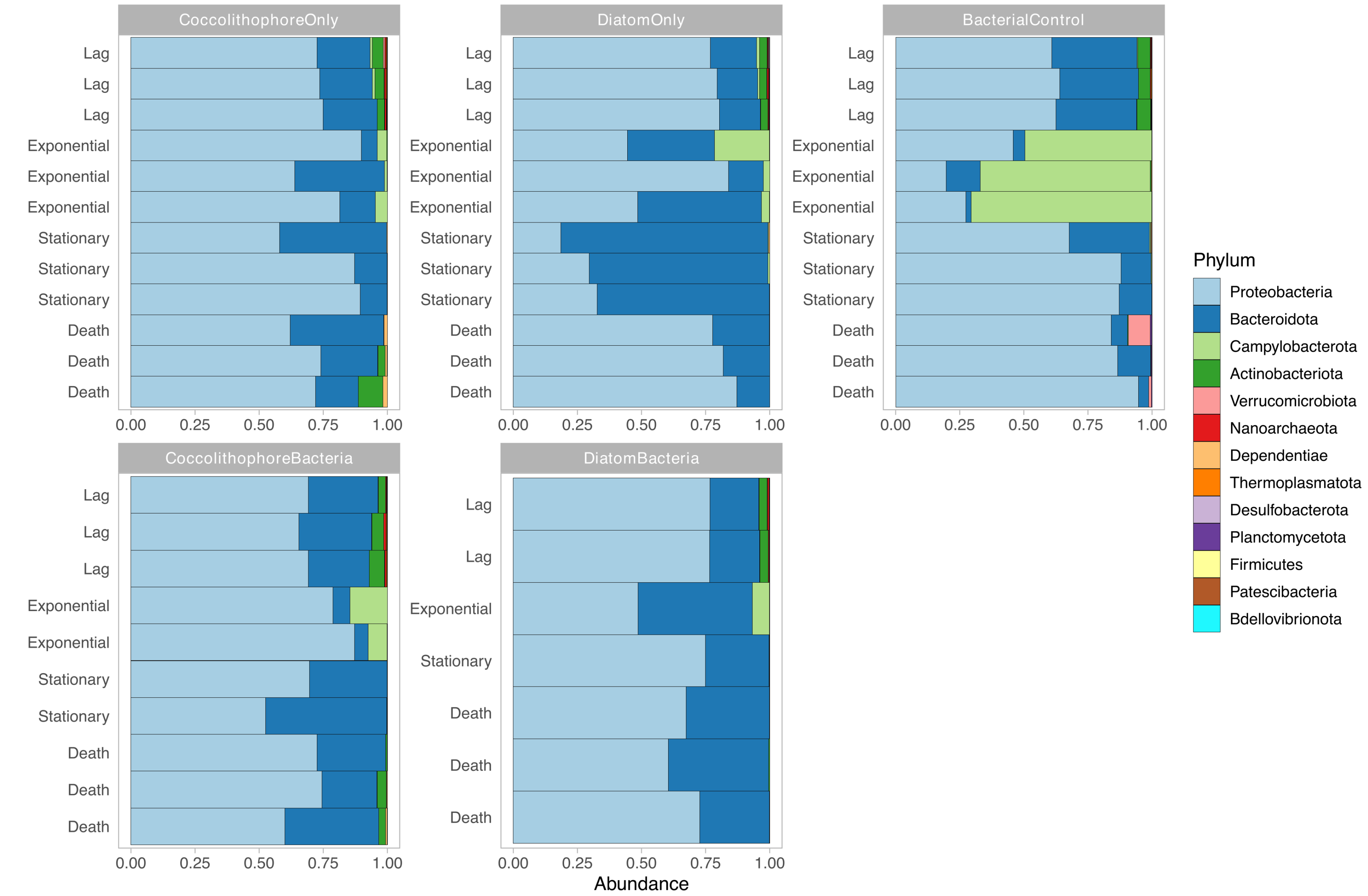

Figure S9 Significantly affected taxa in response to culture conditions grouped at Phylum level. Taxa were identified using a Kruskal-Wallis test for each growth phase separately by comparing control profiles to those with phytoplankton present. Values represent

relative abundance based on shown taxa only.

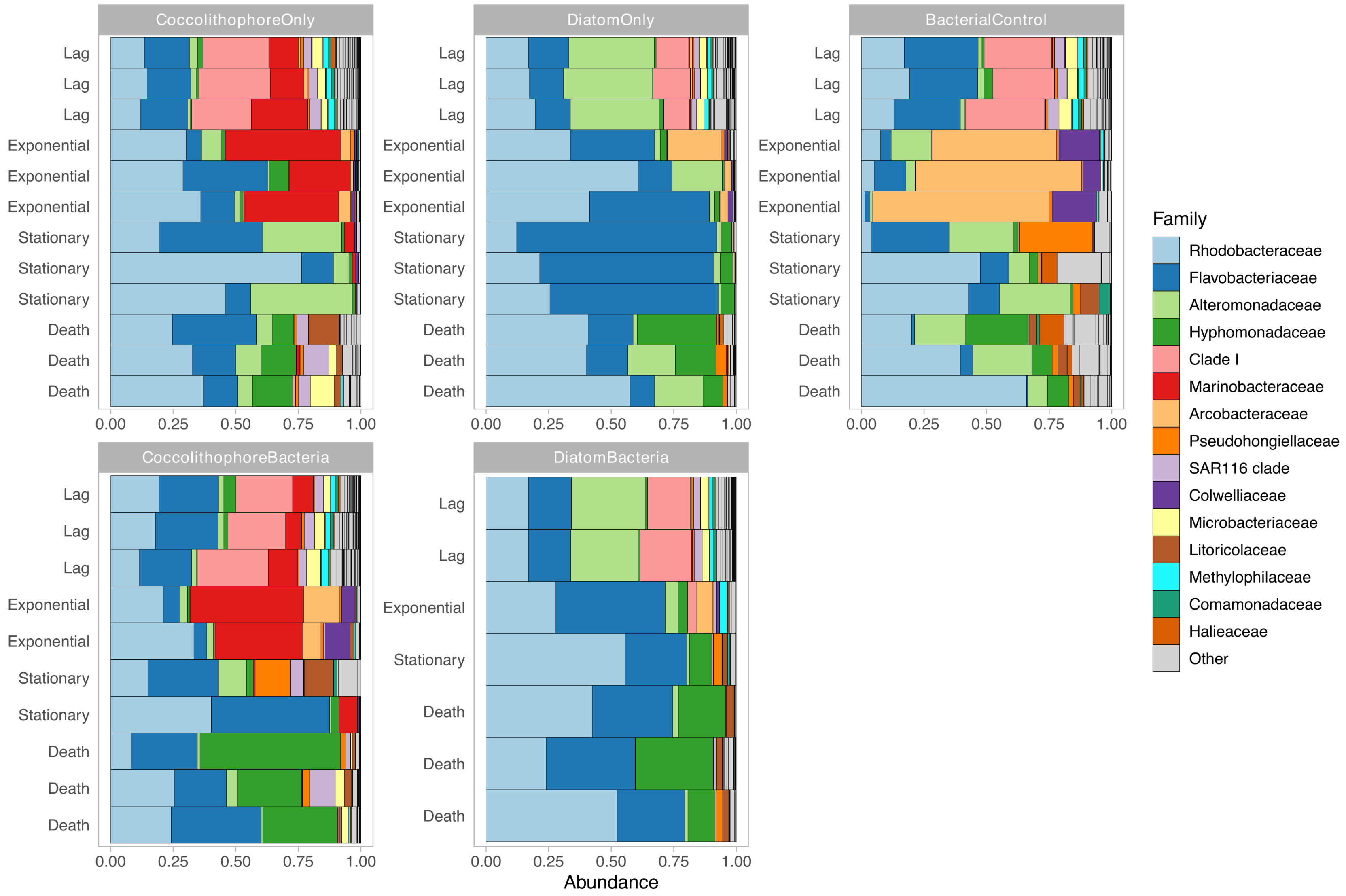

Figure S10 Significantly affected taxa in response to culture conditions grouped at Family level. Taxa were identified using a Kruskal-Wallis test for each growth phase separately by comparing control profiles to those with phytoplankton present. Values

represent relative abundance based on shown taxa only.

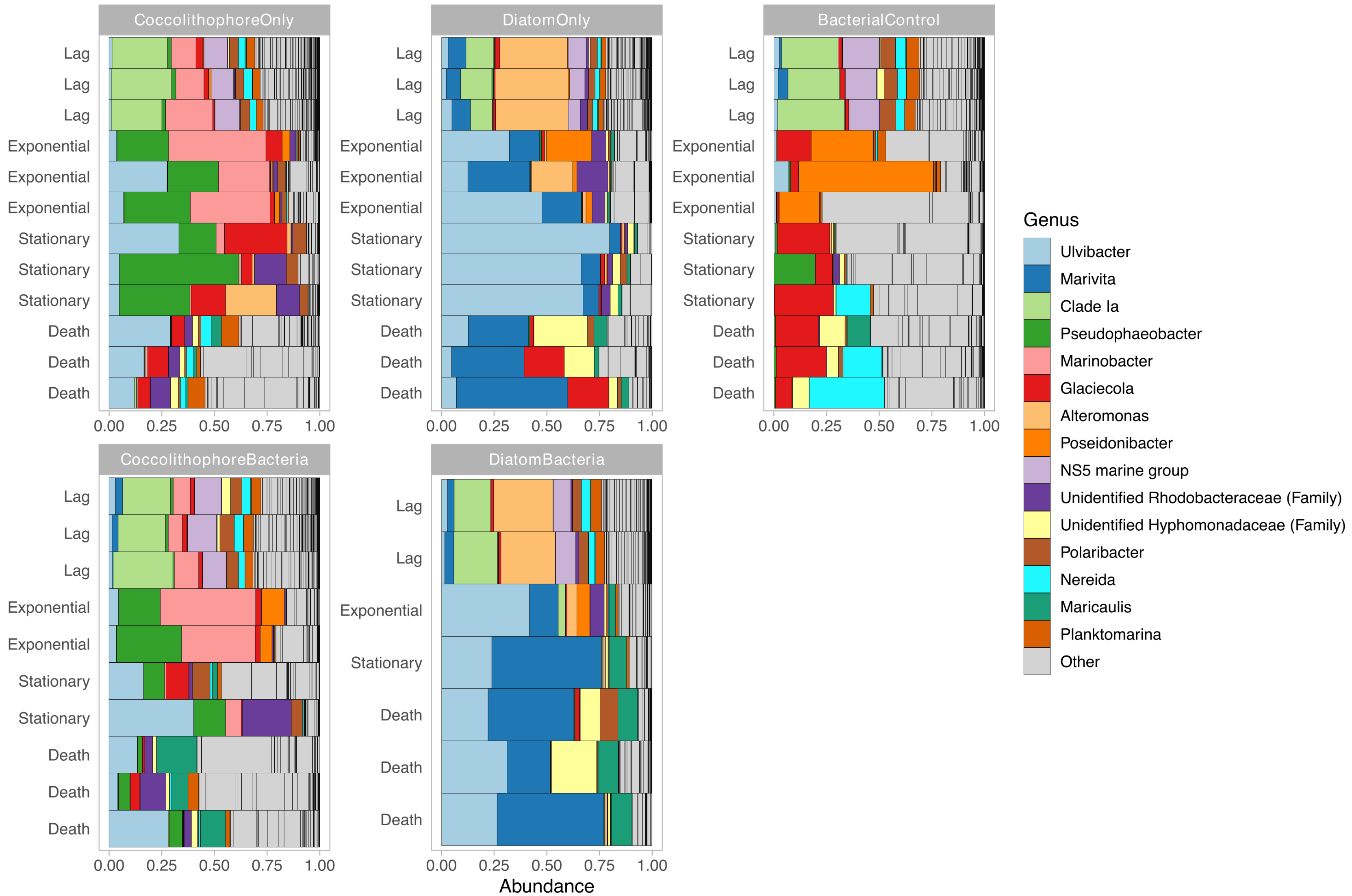

Figure S11 Significantly affected taxa in response to culture conditions grouped at Genus level. Taxa were identified using a Kruskal-Wallis test for each growth phase separately by comparing control profiles to those with phytoplankton present. Values represent

relative abundance based on shown taxa only.

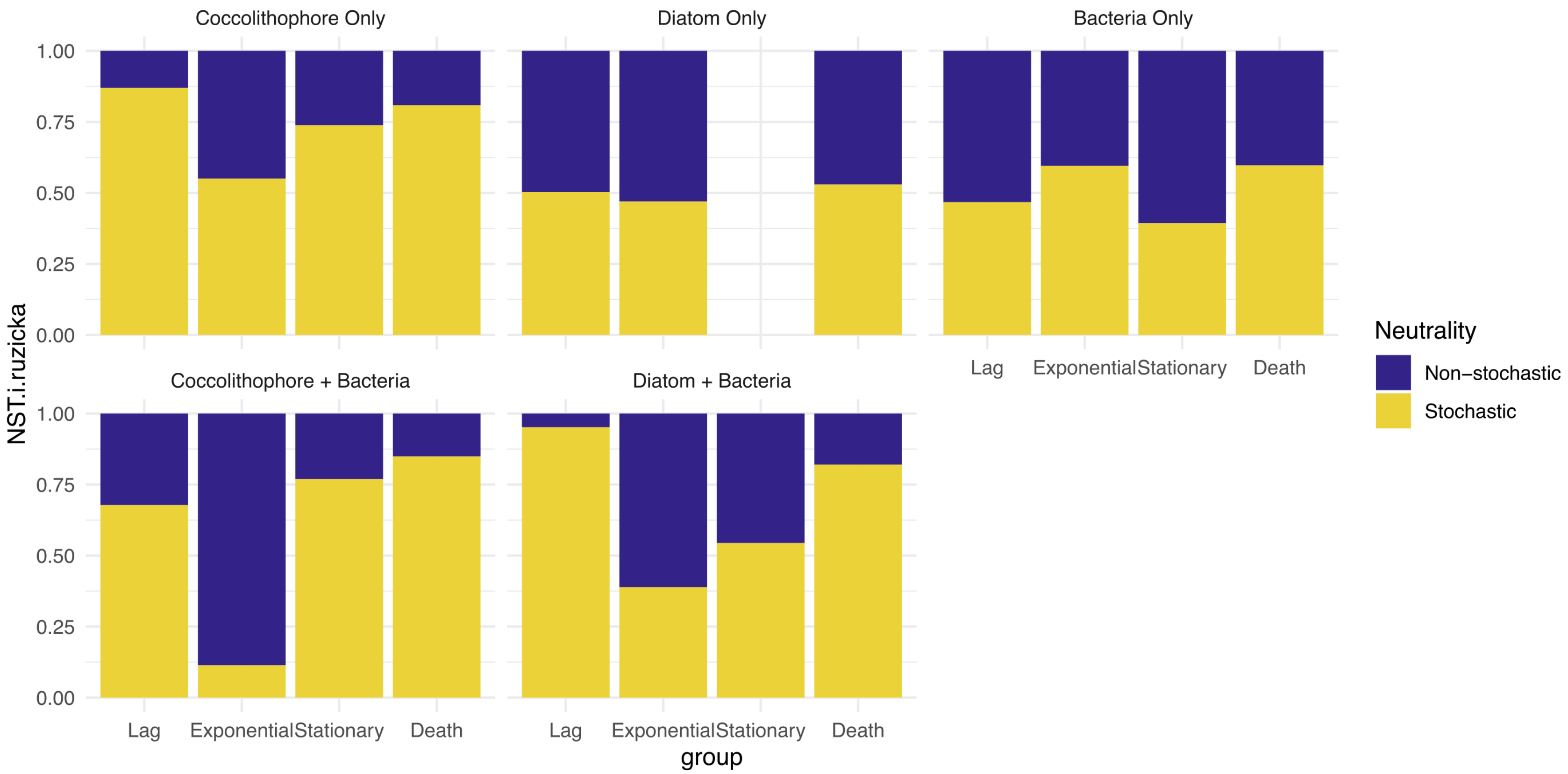

Figure S12 Changes in estimated normalized stochasticity ratio (NST) across culture conditions throughout the phytoplankton growth phases. NST was calculated based on Ružička metrics.

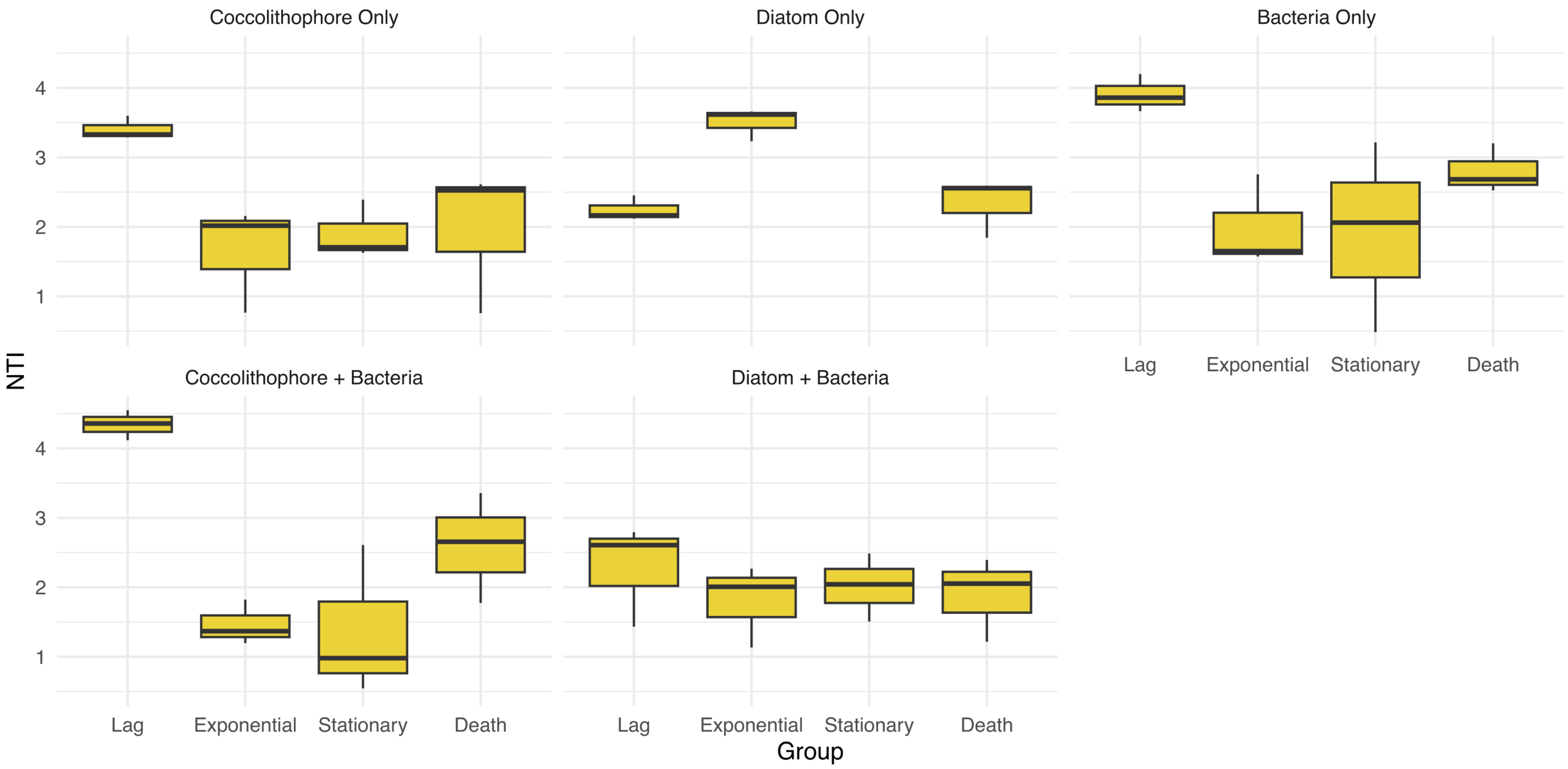

Figure S13 Nearest taxon index (NTI) values calculated with the NTI.p R function across culture conditions throughout the phytoplankton growth phases.
